# Supplementary material for: Biological Redox Impact of Tocopherol Isomers Is Mediated by Fast Cytosolic Calcium Increases in Living Caco-2 Cells
Source: Antioxidants (Basel). 2020 Feb 14;9(2):155. doi: 10.3390/antiox9020155 (PMC7070868; doi:10.3390/antiox9020155)
Supplement: Supplementary file 1 [file antioxidants-09-00155-s001.docx]

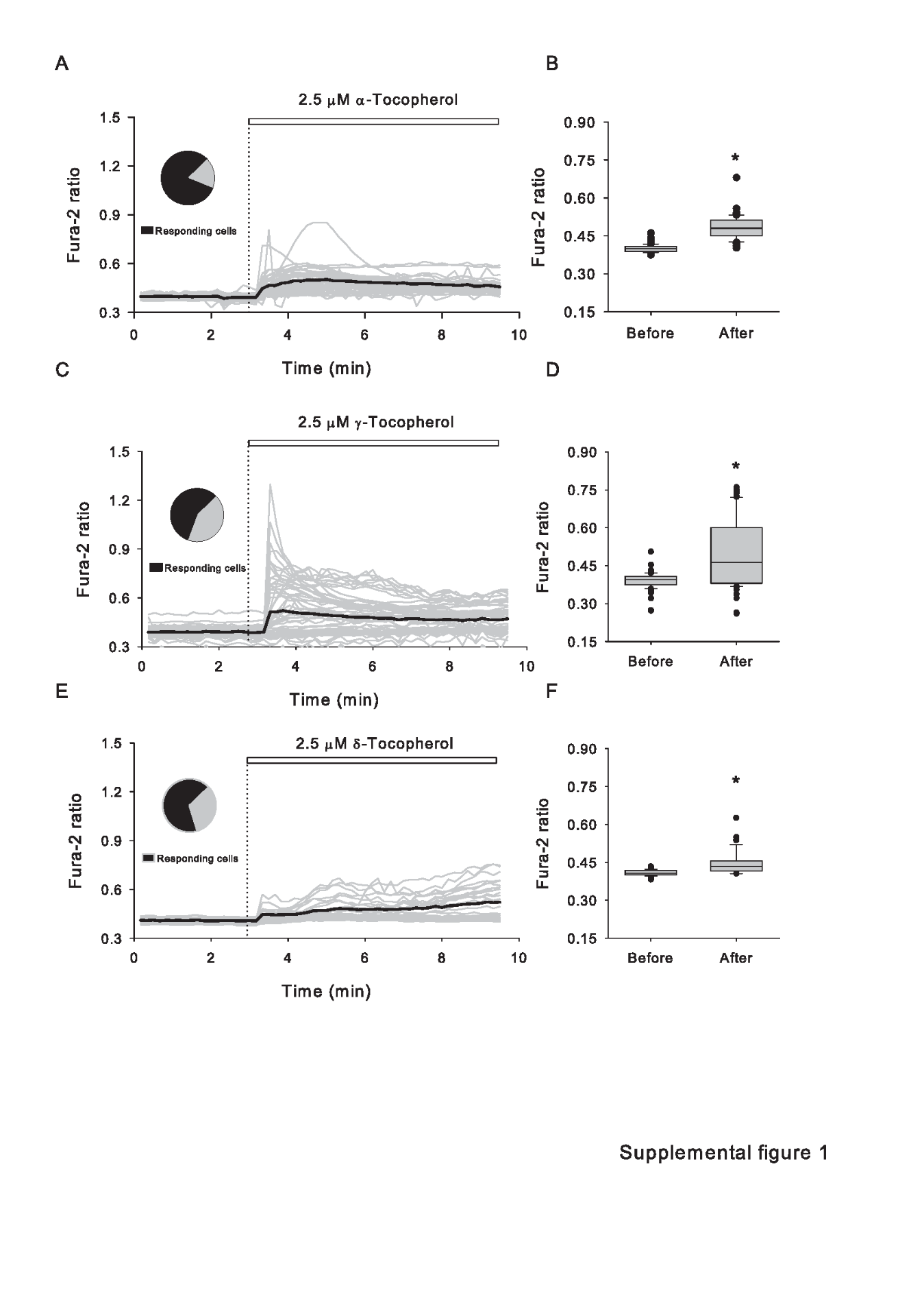


**Figure S1. α-, γ- and δ-tocopherol isomers induce acute cytosolic Ca^2+^ increases at 2.5 μM in Caco-2 cells. (A) Time-course of Fura-2 fluorescence ratio in Caco-2 cells; grey traces in the graph correspond to 67 single-cell recordings from three independent experiments, whereas the black line shows their average. The moment of 2.5 μM α-tocopherol addition is indicated by a dotted line and by the white bar on the plot. The pie chart illustrates the proportion of responding cells in black. (B) Quantification of fluorescence ratio values taken from a bin of 3 min before and after exposure to 2.5 μM α-tocopherol; the line into the boxes corresponds to the median. (C) Same as described in (A), but this graph shows the effect of 2.5 μM γ-tocopherol obtained from 61 single-cell recordings of three independent experiments. The pie chart illustrates the proportion of responding cells in black. (D) Fura-2 ratio values obtained as described in (B). (E) Same as described in (A) and (C), but this graph shows the effect of 2.5 μM δ-tocopherol obtained from 37 single-cell recordings of three independent experiments. The pie chart illustrates the proportion of responding cells in black. (F) Analysis of ratio fluorescence values obtained before and after application of δ-tocopherol as described in (B) and (D). Asterisks indicate significant differences obtained by paired Student’s t-test.**


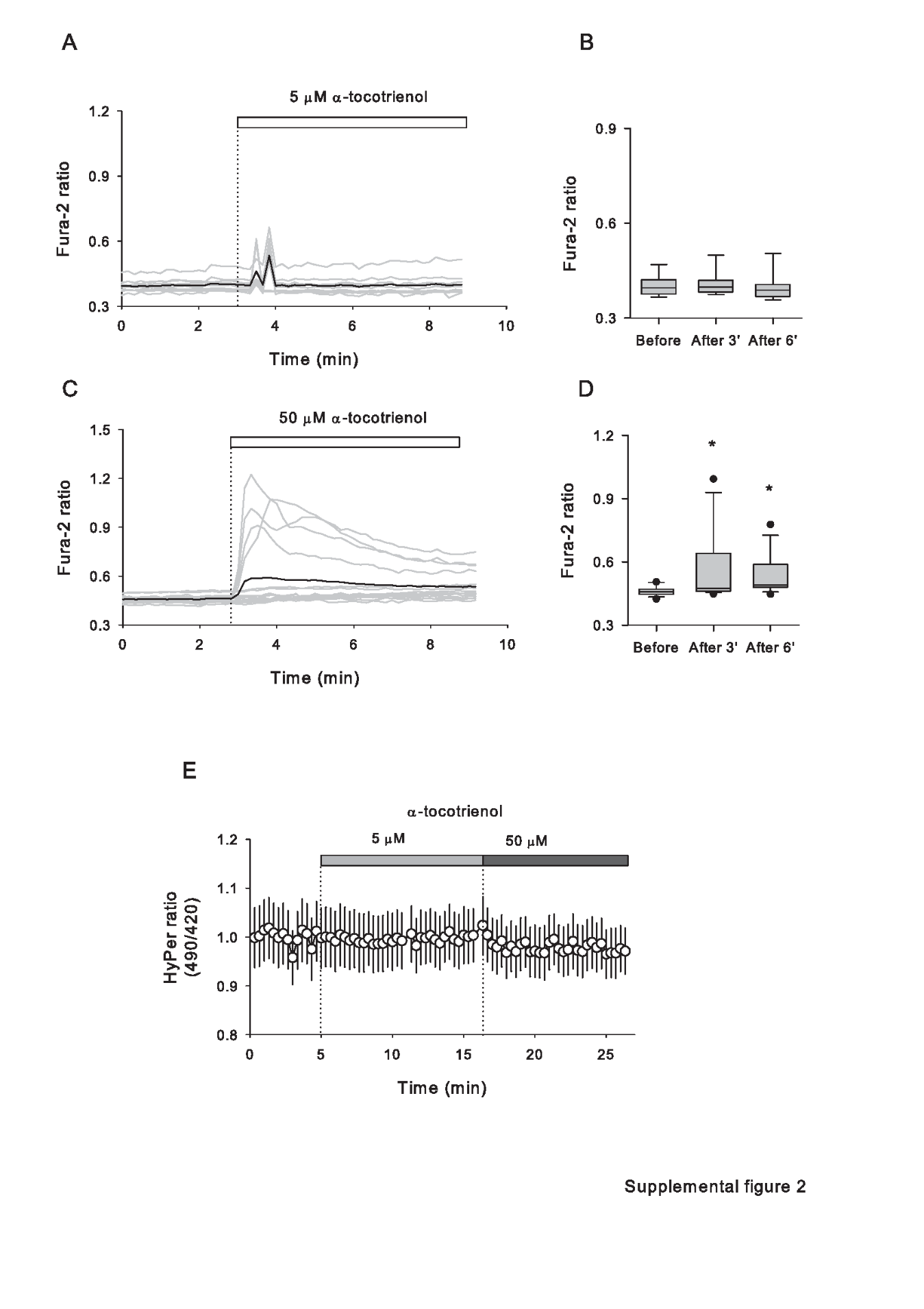


**Figure S2. Time-course of α-tocotrienol exposure on cytosolic Ca^2+^ levels and HyPer signal in Caco-2 cells. (A) Caco-2 cells loaded with Fura-2 were exposed to 5 μM α-tocotrienol at the time indicated by the white bar and the dotted line on the plot. Grey traces in the graph correspond to 19 single-cell recordings from three independent experiments, the black line shows their average. (B) Quantification of fluorescence ratio values taken from a 3 min bin before and after the addition of α-tocotrienol (3 and 6 minutes); the line into the boxes corresponds to the median. (C) Same as described in (A), but this graph shows the effect of 50 μM α-tocotrienol obtained from 17 single-cell recordings of three independent experiments. (D) Fura-2 ratio values obtained as described in (B). (E) Time-course of HyPer signal showing the effect of two consecutive applications of 5 and 50 μM α-tocotrienol obtained from 10 single-cell recordings of four independent experiments. The time of α-tocotrienol addition is indicated by dotted lines and grey bars above the graph. Asterisks mean significant differences between groups according to RM-ANOVA against the control group (Dunn’s method).**


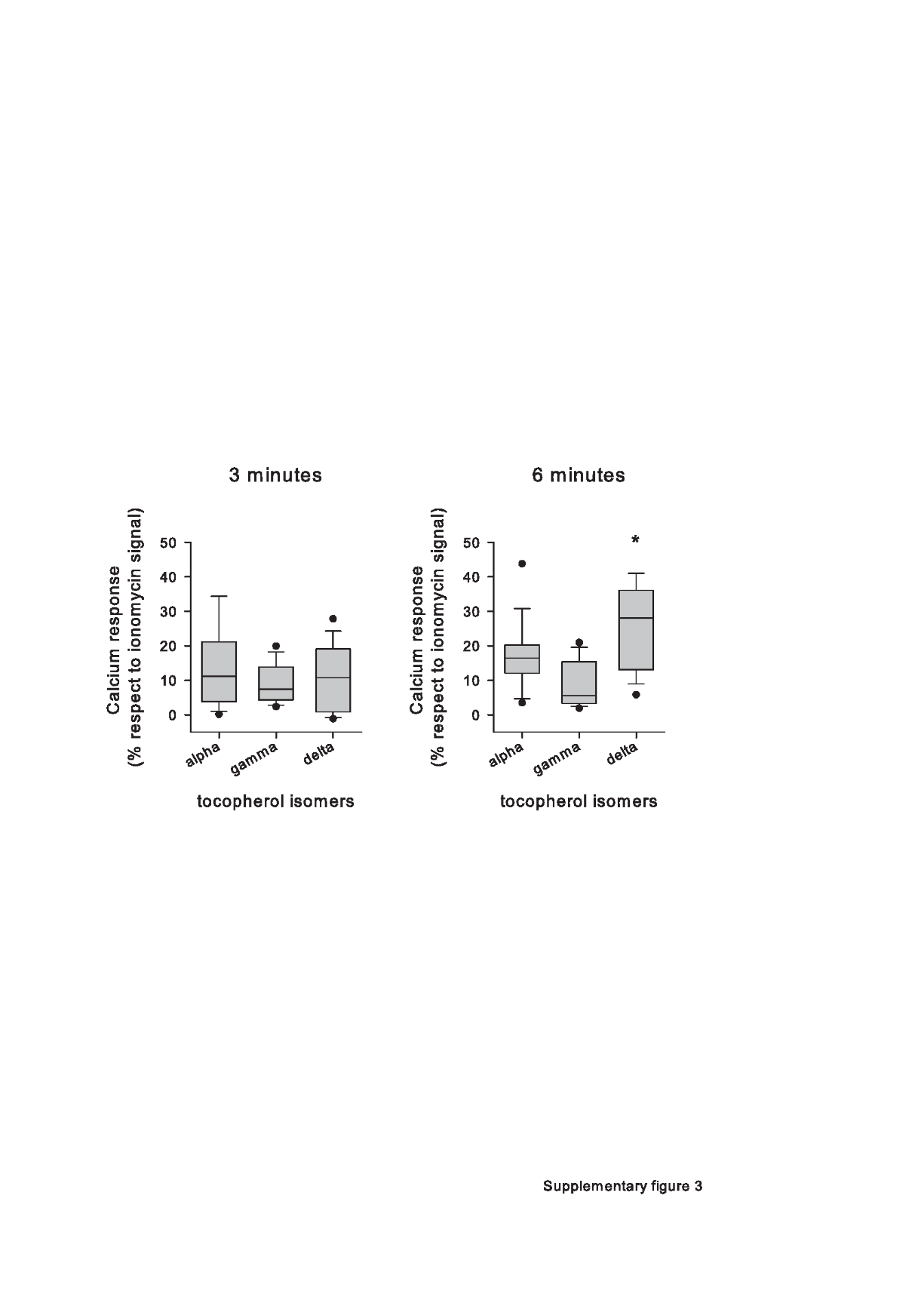


**Figure S3. Comparison of the magnitude of calcium responses induced by α-, γ- and δ-tocopherol at 3 and 6 minutes of exposure. Calcium responses for each single-cell recordings were expressed as the percentage according to the equation % = (r_tocopherol_ – r_basal_)/( r_ionomycin_ – r_basal_) × 100, where r_tocopherol_ corresponds to the average values for Fura-2 ratio obtained at 3 (left) or 6 (right) minutes of isomers exposure; r_basal_ corresponds to the ratio values obtained before any treatments and r_ionomycin_ corresponds to the maximal signal induced by the addition of 2 μM ionomycin, which occurs at the end of each experiment. Number of single-cell recordings and independent experiments are identical to mentioned in the Figure 1.**


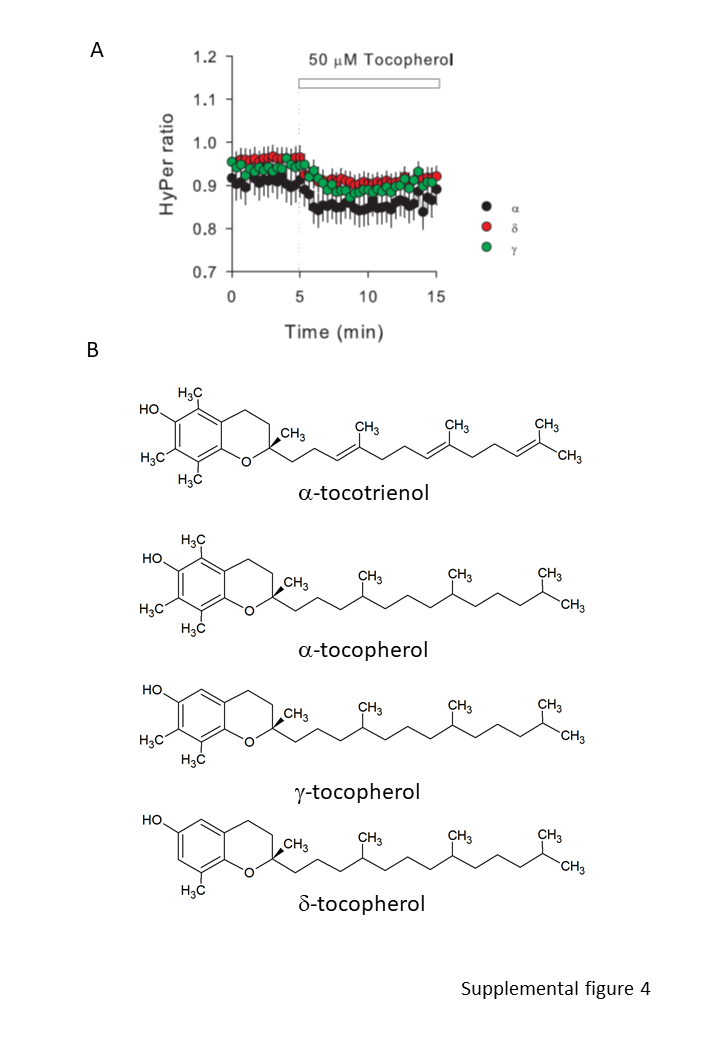


**Figure S4. α-, γ- and δ-tocopherol isomers elicit similar effects on the HyPer biosensor at 50 μM in Caco-2 cells. (A), Time-course of HyPer fluorescence ratio was measured in Caco-2 cells exposed to 50 μM of tocopherol isomers at the time indicated by a dotted line and a white bar on the graph. Data for α-tocopherol are represented by filled circles (29 cells), γ-tocopherol by green circles (32 cells) and δ-tocopherol by red circles (21 cells), all of them obtained from three independent experiments. Data correspond to the average ± SE. (B) Below the plot, the chemical structures for α-tocotrienol and α, γ and δ-tocopherols are shown.**
